# Knowledge and perception of community about causes of cattle abortion and case‐control study of brucellosis as cause of abortion in Jimma zone, Ethiopia

**DOI:** 10.1002/vms3.600

**Published:** 2021-08-14

**Authors:** Dereje Tulu Robi, Benti Deresa Gelalcha, Feyissa Begna Deresa

**Affiliations:** ^1^ Ethiopian Institute of Agricultural Research Tepi Agricultural Research Center Tepi Ethiopia; ^2^ School of Veterinary Medicine College of Agriculture and Veterinary Medicine Jimma University Jimma Ethiopia

**Keywords:** abortion, brucellosis, cattle, Ethiopia, leptospirosis

## Abstract

**Background:**

Abortion, causes by several infectious and non‐infectious factors is one of the most critical health problems of cattle in Ethiopia. Thus, this study aimed to assess knowledge and perception of the community about potential causes of abortion and case‐control study of brucellosis as a cause of abortion in cattle of study districts.

**Methods:**

A cross‐sectional study was conducted between October 2016 and October 2017 in selected districts of the Jimma zone using a questionnaire survey. Based on the questionnaire survey, we also carried out a case‐control study (141 cases and 282 controls) to confirm whether the most frequently mentioned disease (brucellosis) is associated with abortion. The blood samples were collected from both cases and controls cattle groups and then serum was separated. The presence of antibody against *Brucella* organism in serum was first tested by Rose Bengal Plate Test (RBPT) and all RBPT positive samples were confirmed using the Complement Fixation Test (CFT).

**Results:**

From a total of 180 randomly selected respondents, the majority (59.4%) of them attributed abortion to infectious diseases. Based on the questionnaire survey, brucellosis, leptospirosis and listeriosis were identified as the major infectious causes of abortion in the areas. Physical injuries, shortage of feed and toxic substances were observed as less important non‐infection causes of abortion in the study areas. This study also identified improper disposal of aborted materials and birth products, use of communal bulls, sharing communal grazing areas and water sources which favour disease transmission. Exposure to *Brucella* organism was higher among cases (6.4%) than controls (2.8%) with a statistically significant difference (*p* = 0.042).

**Conclusion:**

Brucellosis is the most important infectious cause of cattle abortion in this study. Thus, important to conduct appropriate control methods and increasing public awareness of the zoonotic transmission of brucellosis are suggested. This finding also recommended the need for further study to isolate and characterise brucellosis as a cause of abortion in cattle.

## Graphical abstract

Abortion, caused by several infectious and non‐infectious factors, is one of the most important health problems of cattle in Ethiopia. A cross‐sectional and case‐control study was conducted between October 2016 and October 2017 in selected districts of Jimma zone using a questionnaire survey. Infectious diseases such as brucellosis, leptospirosis, and listeriosis were identified as the major cause of abortion in the areas.

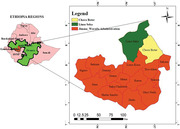

## 1 INTRODUCTION

Ethiopia has paid considerable attention to improve cattle productivity (meat and milk). Breeding and health interventions are the major strategies to be employed to increase the contribution of cattle to economic growth as well as to meet the increasing local demands (Zegeye, 2003). The priority focus area of the dairy development in the country is to develop dairying at the farmer level to increase the supply of milk from smallholder dairy farms (Dessalegn et al., 2016; Tulu et al., 2018). However, reproductive health problems are the major factor that hinders the dairy industry development in the country (Adane et al., 2014; Benti & Zewdie, 2014). Abortion and premature expulsion of the fetus is the main constraint in dairy sector development to achieve its goal (Ernest, 2009; Lobago et al., 2006; Peter, 2000).

The cause of abortion in cattle is multifactorial, which could be infectious and non‐infectious agents (Hovingh, 2009). The infectious agents that cause abortion in cattle include viruses (*Bovine herpes virus 1*, *Bovine viral diarrhea virus*, *Bluetongue virus*, *Schmallenberg virus* and others), bacteria (*Brucella abortus, Coxiella burnetii, Leptospira, Listeria monocytogenes* and others), protozoa (*Toxoplasma gondii, trichomonas* and others) and fungus (*Aspergillus fumigatus*, *Morteriella wolfii* and *Mucor spp*) (Pal et al., 2016; Tulu et al., 2018). Non‐infectious factors such as heat stress, production stress, seasonal changes, chemical poisoning, drug, hormones, nutritional deficiencies and genetic disorders have been reported in some investigations (Hansen, 2002; Regassa & Ashebir, 2016; Sani & Amanloo, 2007). Although the infectious cause of abortion is documented to be the most common cause, diagnosis of abortions a very challenging task both to the veterinarian and cattle owner. Abortion has a direct impact on the reproductive performance of cattle (De Vries, 2006; Dinka, 2013; Hossein‐Zadeh, 2013).

In Ethiopia, studies have shown that abortion in cattle is the most common reproductive health problem (Benti & Zewdie, 2014; Regassa & Ashebir, 2016; Tesfaye & Shamble, 2013). Moreover, abortion results in high economic loss because of reduced milk yield, rebreeding cost and replacement costs to the owners (Carpenter et al., 2006; Peter, 2000; Thurmond et al., 2005). A prevalence as high as ranging from 2% to 29% has been reported (Eshete & Moges, 2014; Gizaw et al., 2007; Siyoum et al., 2016; Tesfaye & Shamble, 2013) in different parts of the country. There is a steady increase in abortion cases of unknown causes in the southwest part of Ethiopia in recent years. Though, no study has been done to identify the potential causes of abortion in the study areas. This study helps to implement appropriate control and prevention methods in the study areas. Therefore, the objective of this study is to assess the knowledge and perception of the community about causes of abortion and case‐control study of brucellosis as the cause of abortion in cattle of Jimma zone, Ethiopia.

## 2 MATERIALS AND METHODS

### 2.1 Description of study areas

**FIGURE 1:**
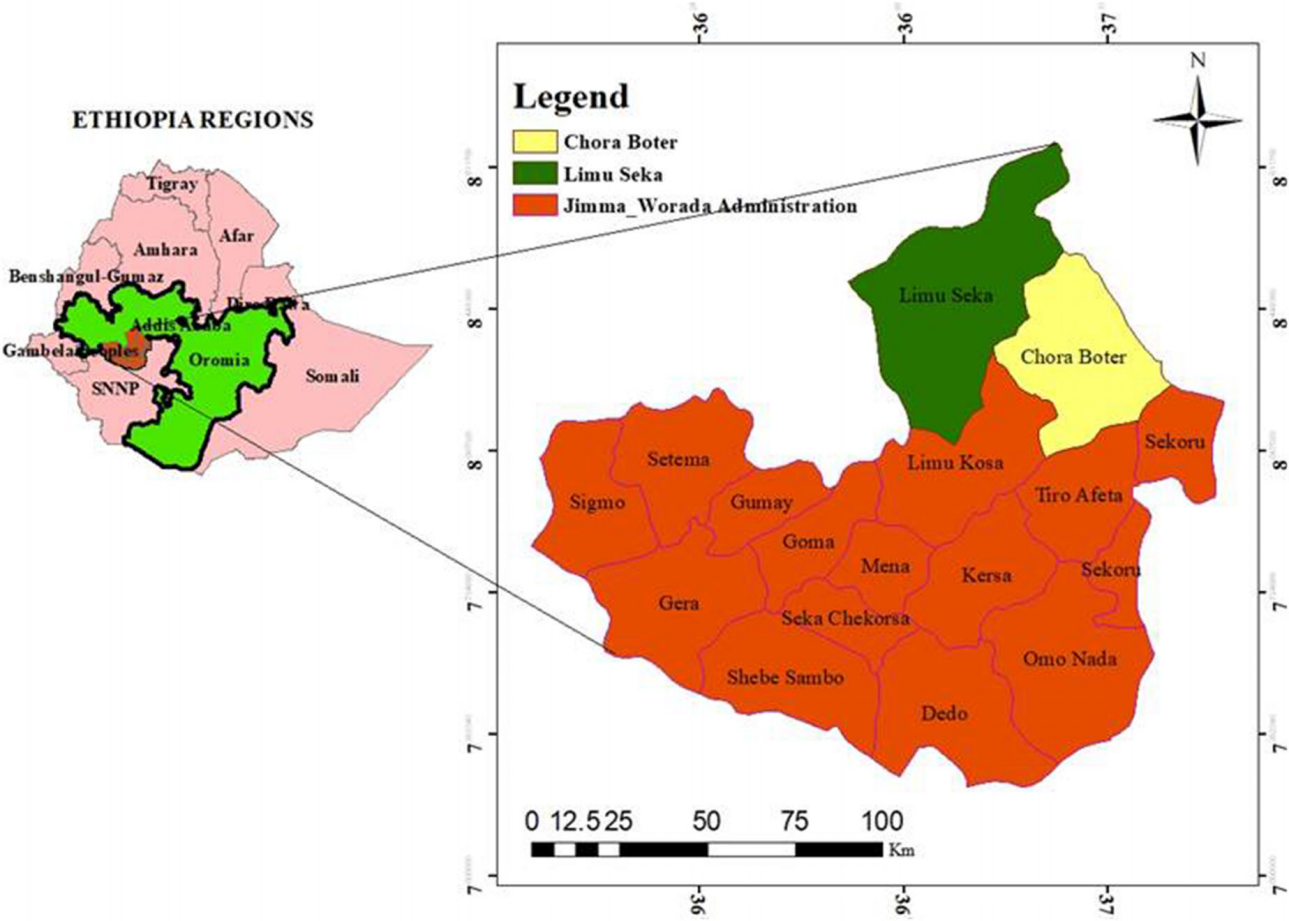
Map of Limu Seka and Chora Boter districts, Jimma Zone, Ethiopia

The study was conducted in the Limu Seka and Chora Boter districts of the Jimma zone. The districts are located at an altitude of 1100–2200 m above sea level, 09°24′ North latitude and 37°56′ East longitudes. The agroecology of the districts is characterised by 19% highland, 64.3% mid‐highland and 17.2% lowland. The annual average temperature ranges from 15.1°C to 31°C. There are two distinct seasons in the districts: the rainy season (from late March to October), and the dry season (November to early March). The rainfall is often more than 1800–2200 mm per annual. The districts have 524,473 cattle, 152, 746 sheep, 157,116 goats and 349,718 human populations (CSA 2017). Local cattle breeds (Horro and Guraghe breeds) are the most dominant ones followed by some crosses of Holstein‐Friesian. The management systems of the districts are extensive (crop‐livestock production) and semi‐intensive (urban production) systems (Figure 1).

### 2.2 Study designs and animals

Cross‐sectional and case‐control studies were undertaken from October 2016 until October 2017 in selected districts of the Jimma zone. A questionnaire survey (using a structured and semi‐structured set of a question) was used to investigate the potential causes of abortion. We carried out a case‐control study to investigate the potential role of brucellosis, the primary disease mentioned as a cause of cattle abortion in the areas. Cows or heifers that had experienced abortion were defined as cases, whereas controls were cows or heifers from the same herd but had no record of abortion. Female cattle with the age of two years and above were included as study animals. Cows or heifers with no history of vaccination against brucellosis were involved in the study. For this study, abortion was defined as the loss of pregnancy at a stage where the expelled fetus is of recognisable size ranging from 42 to 260 days of gestation (Peter, 2000). The *Brucella* organism in cattle indeed causes abortion mainly after the third trimester though it should be suspected if abortion occurs after five months (as the erythritol concentration starts rising during this time). The definition we used is a standard definition of abortion though it is less likely that the owners’ notice early cases of abortion. In our study, ‘a case’ is an abortion that occurred anytime between 42 and 260 days.

### 2.3 Sampling method and sample size determination

We conducted a multistage sampling strategy with the zone as highest and household as lowest sampling stage, district and village in between the two stages. Jimma zone was selected purposively, while districts, peasant associations (the small administrative units in the district), villages and households were selected by a simple random sampling method. Six and four peasant associations were sampled from Limu Seka and Chora Boter districts, respectively based on the number of peasant associations and cattle population. For this study, a total of 180 households were randomly selected from both districts (107 households from Limu Seka and 73 households from Chora Boter) after initially developing of sampling frame from a list of all households in each peasant association of the districts. In the case‐control study, cows/heifers with a history of abortion in the herds were selected purposely based on a case book from veterinary clinics and owners' information. The required minimum sample size was calculated using (Ausvet, 2016) base on a case‐control study design with a predetermined odds ratio (OR) of 3, an expected prevalence of exposure in control groups of 10%, a desired level of confidence 95%, the precision of 5% and a power of 80% (Asmare et al., 2012; Dohoo et al., 2003), thus leading to a sample size of 97 cases. With two controls selected per case, the number of controls should have been 194. The sample size was multiplied by the design effect (D) to correct the variation in design, which was calculated using this formula *D* = ρ (*n* – 1) + 1, where n was an average number of cattle in the cluster (herd) (6), an inter‐correlation coefficient of ρ = 0.09 was described for *Brucella abortus* in cattle (Otte & Gumm, 1997). The design effect (*D*) was 1.45 and increasing the power by using two controls per case. Thus, a minimum of 141 cases and 282 controls were selected to be enrolled in this study.

### 2.4 Data collection

A total of 180 cattle keepers were randomly selected and interviewed. To get information on the potential cause of abortion, gestation stage of abortion and management of abortion. In addition, some variables such as grazing system, water source, breeding system and feeding of storage feed also recorded. The disposal of abortion and birth product also studied. The questionnaire was administered using face‐to‐face interviews of respondents. In the case‐control study, the important information related abortion history of animals was also recorded.

### 2.5 Sample collection and serological tests

About 10 ml blood samples were collected from the jugular vein of each cattle by using a sterile needle and plain vacutainer tube. Identification of each cattle was labeled on corresponding vacutainer tube and blood samples were allowed to stand overnight at room temperature to obtain the serum. The animals’ codes were transferred to the cryovials to which the serum was decanted and serum samples were kept at –20°C (OIE, 2009) in Jimma University microbiology laboratory until they transported to National Veterinary Institute, Debre Zeit using an icebox for serological analysis. The serum samples were screened by using Rose Bengal Plate Test (RBPT) (KT153NB, UK) for the presence of *Brucella* agglutinins according to OIE (OIE, 2004) procedures. The serum samples and antigens were taken from the refrigerator, and then it stays at room temperature for half an hour and processed following the recommended procedures. The interpretation of both positive and negative control results was done according to the degree of agglutination and the reaction was read in a good light source or a magnifying glass when micro agglutination was suspected. The RBPT results were interpreted 0, +, ++ and +++ as described by (Dohoo et al., 2009), where 0 indicated no agglutination, + indicated barely visible agglutination (using magnifying glass), ++ indicated fine agglutination and +++ indicated coarse clumping. Those serums indicated with no agglutination (0) were regarded as negative, while those with +, ++ and +++ were considered as positive. All RBPT positive serum was further tested using a complement fixation test (CFT) using standard *B. abortus* antigen S99 and control sera (positive and negative) (KT15 3NB, UK). Reagent making was estimated by titration and done according to the procedure recommended by OIE (OIE, 2004).

### 2.6 Data management and analysis

Data recorded from this study were stored in Microsoft Excel for Windows 2010 and transferred to SPSS version 20.0 (IBM SPSS, 2011). Descriptive and analytical statistics for most variables were analysed using SPSS software. Associated between the history of abortion and explanatory variables was tested using χ^2^ test. The Student's *t*‐test was conducted to compare the mean cattle population in study areas. Association between abortion and seroprevalence of brucellosis was carried out using *Z*‐test. The units of analysis were individual cattle, herd and interviewed households. Confidence intervals (CI) 95% and *p* ≤ 0.05 were set for significance in all analyses.

## 3 RESULTS

### 3.1 Cross‐sectional study

The age of respondents ranged between 22 and 76 years with an average mean age (mean ±SD) of 47.99 ±15.83 years. The majority of the interviewees (78.9%) were males. The majority of the respondents (57.8%) in the study areas had attended elementary school (Table 1).

**TABLE 1:** Socio‐demographic characteristics of respondents in study areas

Parameters	Limu Seka (*N* = 107)	Chora Boter (*N* = 73)	Overall (*N* = 180)
Age of the respondents^a^	48.35 ± 15.89	47.48 ± 15.83	47.99 ± 15.83
Sex of the respondents^b^			
Male	91 (85.0)	51 (69.9)	142 (78.9)
Female	16 (15.0)	22 (30.1)	38 (21.1)
Educational level^b^			
Illiterate	36 (33.6)	10 (13.7)	46 (25.6)
Read and write	13 (12.1)	6 (8.2)	19 (10.6)
Elementary (1–8)	51 (47.7)	53 (72.6)	104 (57.8)
Secondary school (9–12)	5 (4.7)	4 (5.5)	9 (5.0)
College graduate	2 (1.9)	0 (0.0)	2 (1.1)

*N* = number of respondents.

^a^Value given as mean ± SD.

^b^Value given as *N* (%).

This result revealed a statistically significant difference (*p* < 0.05) between the study areas in the size of heifers and oxen. There were a significantly larger number of heifers (2.88 ± 0.40) in Chora Boter than Limu Seka (2.15 ± 1.39) districts. The average size of oxen (5.88 ± 1.42) in Chora Boter district was larger than in Limu Seka districts (4.02 ± 1.79) (Table 2).

**TABLE 2:** Cattle herd structure in the study areas (Mean ± SE)

				Test	
Herd type	Limu Seka (*N* = 107)	Chora Botor (*N* = 73)	Overall (*N* = 180)	*t* Value	*p* Value
Milking cows	4.47 ± 1.05	4.90 ± 1.39	4.64 ± 1.20	1.31	0.192
Pregnant cows	2.17 ± 1.31	2.47 ± 1.49	2.29 ± 1.39	1.41	0.160
Dry cows	0.29 ± 0.05	0.0 ± 0.0	0.22 ± 0.03	1.49	0.167
Heifers	2.15 ± 1.39	2.88 ± 0.40	2.37 ± 1.99	4.35	0.001*
Male calves	2.68 ± 1.23	3.27 ± 1.66	2.92 ± 1.43	1.61	0.109
Female calves	2.26 ± 1.71	2.40 ± 1.73	2.32 ± 1.72	0.52	0.605
Bulls	0.59 ± 0.11	0.0 ± 0.0	0.67 ± 0.47	1.63	0.105
Oxen	4.02 ± 1.79	5.88 ± 1.42	4.77 ± 3.65	3.46	0.001*

* Significant.

In this study, about 62.3% of the respondents did not take the aborted cattle to a veterinary clinic. The majority of the respondents (59.4%) bred their cattle using common bull, while 33.9% and 6.7% of them used their own bull and AI, respectively. About 71.1% of respondents in study areas used common grazing land. The primary water source for cattle in the study areas was spring water (80%). Most of the respondents (66.7%) conserved feed for their cattle in their backyard. Those variables were significantly associated (*p* < 0.05) with the occurrence of abortion in cattle (Table 3).

**TABLE 3:** Associated risk factors of cattle abortion in the study areas

Parameters	Limu Seka (*N* = 107)	Chora Boter (*N* = 73)	Overall (*N* = 180)	χ^2^	*p* Value
Measure taken during abortion					
Take to veterinary clinic	34 (31.8)	33 (45.2)	67 (37.2)	55.60	0.034
Do not take to veterinary clinic	73 (68.2)	40 (54.8)	113 (62.3)		
Breeding system					
Own bull	61 (57.0)	0 (0)	61 (33.9)		
Common bull	36 (33.6)	71 (97.3)	107 (59.4)	74.00	0.001
Artificial insemination	10 (9.3)	2 (2.7)	12 (6.7)		
Grazing system					
Communal	74 (69.2)	54 (74.0)	138 (71.1)		
Private	23 (21.5)	14 (19.5)	37 (20.6)	66.50	0.039
Both	10 (9.3)	5 (6.8)	15 (8.3)		
Water source					
River	10 (9.3)	13 (17.8)	23 (12.8)		
Pond	9 (8.4)	3 (4.1)	12 (6.7)	79.87	0.007
Spring	88 (82.2)	57 (78.1)	145 (80.6)		
Feed storage					
Yes	71 (66.4)	49 (27.2)	120 (66.7)	48.40	0.038
No	49 (32.7)	24 (32.9)	60 (33.3)		

*N* = number of respondents.

A good proportion of respondents (31.7%) reported one or more cases of abortion in their herd in the past 1 year. Twenty‐eight percent of those surveyed reported that retained fetal membrane was occurred followed by abortion. The majority of the respondents (56.7%) were not aware that some causes of abortion could be transmitted to other animals. In addition, most (76.1%) of the respondents were not knowledgeable about the potential transmissibility of some abortifacient agents to a human. Thus a good proportion (66.1%) of respondents did not handle and dispose of aborted materials or birth products properly (Table 4).

**TABLE 4:** Knowledge and perception of respondents on transmissibility of cause abortion and management of aborted material

	Number of respondent (*N* = 180)	
Parameters	Yes (%)	No (%)
Presence of abortion in herds	57 (31.7)	123 (68.3)
Presence of retained fetal membrane	51 (28.3)	129 (71.7)
Transmissibility of cause of abortion to other animals	78 (43.3)	102 (56.7)
Proper management of aborted material or birth products	61 (33.9)	119 (66.1)
Transmissibility of cause of abortion to human	43 (23.9)	137 (76.1)

*N* = number of respondents.

**FIGURE 2:**
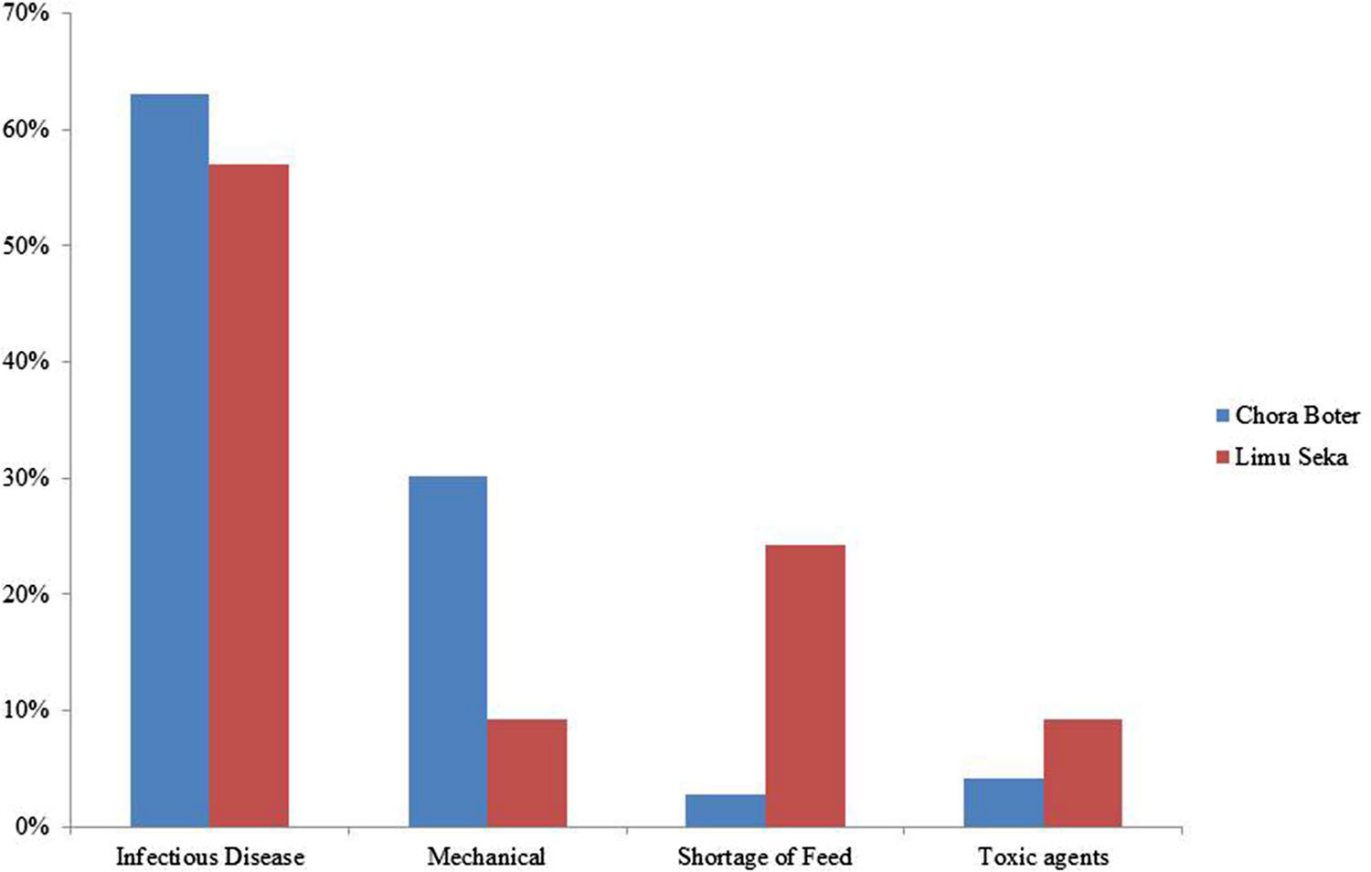
Major causes of abortion as mentioned by respondents in study areas

Infectious agents were mentioned as the most likely cause of abortion by the majority (59.4%) of respondents in study areas (Figure 2). Those causes of abortion were significantly associated (χ^2 ^= 13.87, *p* = 0.003) with the occurrence of abortion in cattle using the χ^2^ test.

**FIGURE 3:**
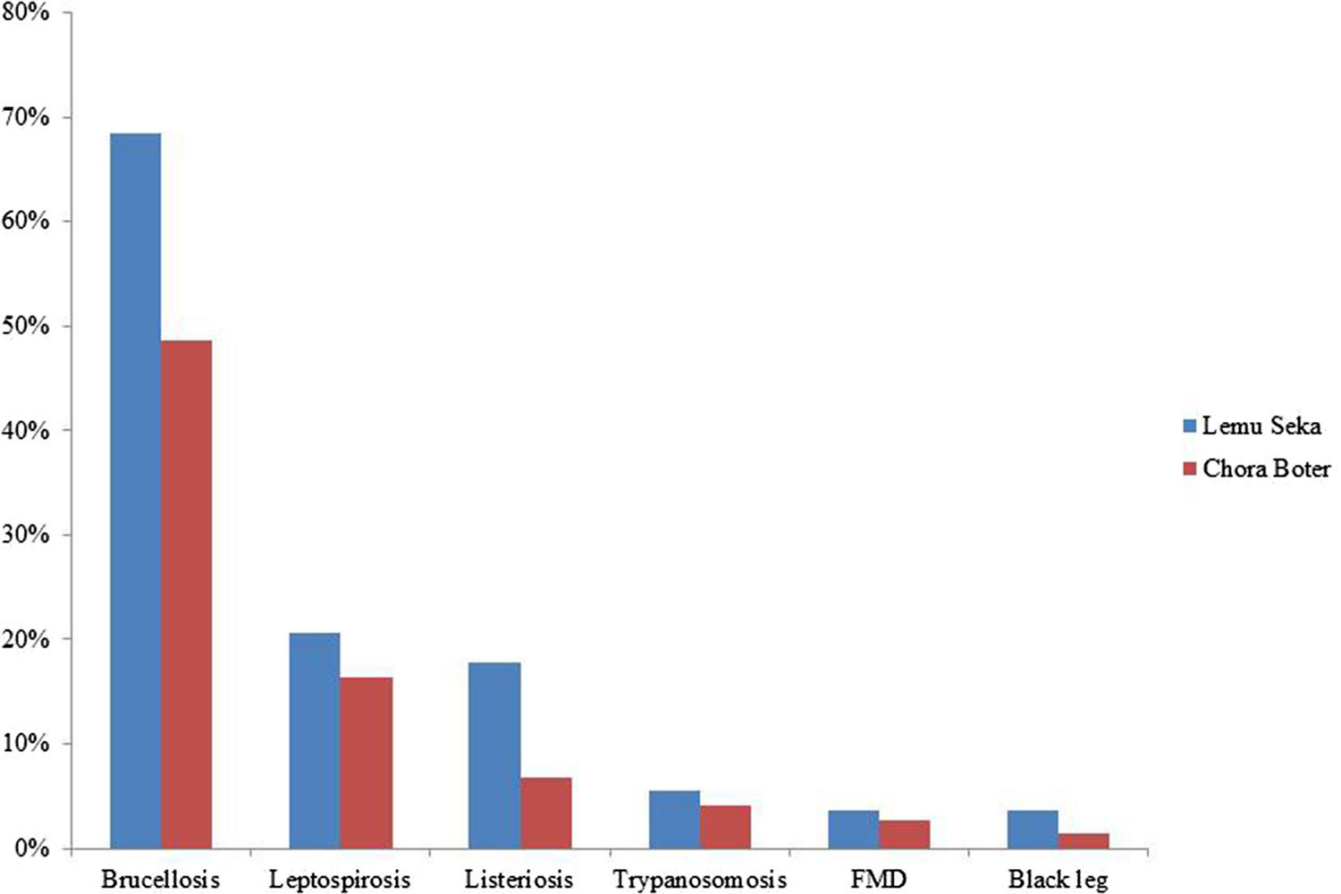
Infectious diseases causes of abortion in study areas

The common infectious causes of abortion in cattle were named in Afan Oromo, the local language. The respondents recognised brucellosis as ‘Gatachiisaa’, characterised by abortion, retained fetal membrane and infertility. Leptospirosis was identified by cattle keeper as ‘Dhukkuba Hantuuta’, described by abortion, coffee/dark coloured urine and yellowish discolouration of the eye. Listeriosis which the farmers locally called ‘Dhukkuba Hokaa’ was mentioned as other causes of abortion in areas. The farmer noted that the disease causes abortion and the cattle to move in circles. Trypanosomosis was known locally as ‘Gowwoomsaa’ which was incriminated as one of the causes of abortion by respondents was associated which the bite of flies and characterised by loss in body weight and loss of tail hair. Foot and mouth disease (FMD) is called locally ‘Maasa’ which is characterised by abortion, salivation, lameness and vesicle on feet and mouth. Blackleg was locally called ‘Gubaa’ which is characterised by abortion, lameness and swelling of the hind leg. Brucellosis (61.1%), leptospirosis (18.9%), listeriosis (12.2%), trypanosomosis (5%), FMD (3.3%) and blackleg (2.8%) were mentioned as the leading infectious diseases causing an abortion in study areas (Figure 3).

### 3.2 Case‐control study

In this study, a total of 423 female cattle (141 cases and 282 controls) were sampled. From a total of 423 tested cattle, 4.5% and 4.1% were positive for *Brucella* antibody by using RBPT and CFT, respectively. A higher seroprevalence of brucellosis was observed in cases (6.4%) than controls (2.8%). A statistically significant difference was observed between cattle abortion and seroprevalence of brucellosis (*p* < 0.05) in study areas (Table 5).

**TABLE 5:** Brucella antibody determined from cases and controls female cattle of the study areas

Brucellosis status	Cases	Controls	Total	*Z* test (*p* value)
Positives	9	8	17 (52.9%)	0.042
Negative	132	274	406 (32.5%)	
Total	141 (6.4%)	282 (2.8%)	423 (4.1%)	

## 4 DISCUSSION

The average size of cows and heifers kept by the farmers was higher than the rest of the cattle classes. Ownership of cows and heifers are differed significantly (*p* = 0.001) between study areas. This may indicate that farmers in the study areas keep cattle mainly for milk production and heifers were used for replacement purposes. A similar finding was reported by (Berihu & Toffik, 2014; Mengistu et al., 2017) where heifer and cows dominate their herd composition followed by oxen. The oxen were important for plowing land for crop cultivation and also for threshing.

The present study indicated that abortion (31.7%) was one of the major reproductive health problems. Most respondents indicated that abortion occurred at the last stage of pregnancy in study areas. This finding concurs with the work (Benti & Zewdie, 2014; Dinka, 2013) who reported abortion as one of the major reproductive challenges in central and southern Ethiopia. Similar results have been published by (Dechicha et al., 2010), who reported abortion as one of the main constraints to cattle production in other African countries like Algeria.

The majority of this study participants responded that they did not take aborted cows to a veterinary clinic (*p* = 0.034). Similarly, (Ebrahim et al., 2016) reported that the majority of respondents did not take aborted animals to a veterinary clinic (82.8%) in Kersa district, southwestern Ethiopia. This indicates that abortion is common and is not considered a serious health problem by livestock producers as its effect is subtle. Most of the respondents failed to properly manage abortion products as they simply leave them on the ground or give it to dogs. This practice would favour the transmission of contagious pathogens to susceptible species of animals including human beings. This is particularly true for some hardy pathogens which survive long in the environment. Furthermore, the lack of awareness on potential means of transmission of abortion‐causing pathogens could pose a great risk of the spread of the disease to humans and animals. This finding concurs with the report of (Tolosa, 2004) and (Bashahun et al., 2015) in selected sites of the Jimma zone.

Most of the respondents bred their cattle using common bull (*p* = 0.001). This might increase the chance of transmission of venereal transmitted diseases that causes abortion in cattle such as trichomoniasis and some extent leptospirosis among herds (Ndengu et al., 2017). This finding agrees with previous studies (Anteneh et al., 2010; Ebrahim et al., 2016; Mengistu et al., 2017; Regassa & Ashebir, 2016) that reported most cattle producers in most parts of the country used common bulls for breeding.

Most of the respondents (66.7%) in study areas conserve feed (grass) for their cattle (*p* = 0.038) in their backyard. Although it is a very good strategy, this practice has its own risk as it can harbour some pathogens if it is poorly stored. One of the most important abortifacient pathogens associated with poor feed storage includes *Listeria* and Mycotic microorganisms (Chandranaik et al., 2014). Interestingly, farmers have explicitly described listeriosis (Dhukkuba Hokaa) (with its clinical signs and symptoms) as one of the most important causes of abortion in the study areas. *Listeria* abortions usually develop in the rainy season due to farmers feed stored grass to their dairy cattle. Cattle abortions due to this organism mostly occur in the last trimester of pregnancy (Yaeger & Holler, 2007). The finding concurs with (Radostits et al., 2007), who stated the listeriosis abortion is related to stored forage and silage feeding to cattle.

The majority of the respondents used common grazing land (*p* = 0.039) and water source (*p* = 0.007) for their cattle in study areas. This could increase frequency contact between cattle at common feeding and watering points. This in turn creates a favourable condition for the transmission of infectious causes of abortion among herds. In addition, herding a large number of animals (overcrowding) could also predispose the animals to mechanical causes of abortion as mentioned by respondents (Matope et al., 2010). This finding is in line with the reported of (Ndengu et al., 2017).

The various causes of abortion (infectious diseases, mechanical or physical agents, shortage of feed and toxic agent) mentioned by respondents (*p* = 0.003) in study areas agrees with standard veterinary textbooks and literature (Givens, 2006; Hovingh, 2009; Ortega‐Mora et al., 2007; Pal et al., 2016; Peter, 2000) that reported the occurrence of abortion in cattle could be due to nutritional deficiencies, trauma, toxicities, or infectious agents and most of the abortion in cattle caused by infectious diseases. Most of the respondents suggested that the likely infectious causes of abortion were brucellosis, leptospirosis and listeriosis.

Abortion is one of the characters of leptospirosis (Dhukkuba Hantuuta) in female cattle. Abortion usually occurs during the last trimester of pregnancy but can occur from the 4th month onwards (Radostits et al., 2007). This may be due to *Leptospira* organisms prefer pregnant uterus to proliferate (Yadeta et al., 2016). Similarly, leptospirosis was stated as the most important cause of abortion in cattle in Ethiopia (Yadeta et al., 2016) and elsewhere (Dechicha et al., 2010; Elelu et al., 2016; Ndengu et al., 2017). Leptospirosis has been reported also one of the five priority zoonotic diseases identified in the country (Pieracci et al., 2016).

A good proportion of the respondents indicated that abortion was followed by a retained fetal membrane. Abortion followed by a retained fetal membrane is mainly associated with brucellosis as *Brucella* infection is characterised primarily by a retained fetal membrane in cattle (Radostits et al., 2007). A diseased cow usually aborted between the 5th and 7th month of pregnancy. Abortion caused by brucellosis (Gatachiisaa) commonly occurs during the last trimester of pregnancy (Parthiban et al., 2015). Similar results have been reported elsewhere state that most cattle keepers knew brucellosis as a cause of abortions (Dechicha et al., 2010; Ndengu et al., 2017). Some of the respondents also mentioned that trypanosomosis (Gowwoomsaa) and FMD (Maasa) were associated with abortion in cattle. This might be due to any disease‐causing high fever in cattle that can potentially cause abortion. This does not have to be a disease that affects the reproductive tract (Radostits et al., 2007).

The case‐control study was carried out to verify the disease was primarily incriminated as a cause of abortion by the farmers in the study districts. A statistically significant difference (*p* = 0.042) was observed in the seroprevalence of brucellosis between cases and controls. This suggests that brucellosis may be associated with cattle abortion in study areas. This result confirms the idea of the farmers as they stated brucellosis (Gatachiisaa) was the most cause of cattle abortion in their areas. Even though this result differs from earlier studies (Asmare, 2014; Asmare et al., 2012; Kebede et al., 2008; Shabbir et al., 2013), they are consistent with those of (Adugna et al., 2013; Berhe et al., 2007; Derdour et al., 2017; Haileselassie et al., 2011; Tsegaye et al., 2016). The variation between results may be due to differences in agroecology, breed, management and husbandry condition in the areas. This can also be differences between the study areas regarding conditions that could favour the transmission of the various causes of abortion (Radostits et al., 2007).

## 5 CONCLUSION

The information generated in this study provides some important insights about abortion's potential causes. Brucellosis is the most important infectious cause of cattle abortion in this study. The finding of this study indicated that *Brucella* seropositive status was associated with abortion in cattle. Further intervention is needed to address the risk practices like poor disposal of abortion products, lack of awareness on the causes and transmission of the abortion‐causing pathogen, use of the communal bull, common water source and grazing land. Moreover, the need for implementing control measures, making the public aware of the transmission of brucellosis and further study should be conducted to isolate and characterise brucellosis as the cause of abortion in cattle.

## ETHICS STATEMENT

All procedures were conducted according to the experiment practice and standards approved by the animals' welfare and research ethic committee at Jimma University that is following the international guidelines for animal welfare with AgVmVM/16/1 reference number. Additionally, verbal consent was obtained from owner of the animals. Full cooperation and voluntary participation of all participants was obtained by assuring them the confidentiality of their involvement.

## CONFLICT OF INTEREST

The authors have not declared any conflict of interests.

## AUTHOR CONTRIBUTIONS

Dereje Tulu Robi: Conceptualisation; formal analysis; investigation; methodology; software; writing‐original draft.

### PEER REVIEW

The peer review history for this article is available at https://publons.com/publon/10.1002/vms3.600

## Data Availability

The data that support the findings of this study are available from the corresponding author upon reasonable request.
